# Systematization of perioperative nursing care in robotic surgery: instrument validation

**DOI:** 10.1590/0034-7167-2022-0666

**Published:** 2023-12-08

**Authors:** Laís Vilanova Tavares Vitoriano, Adriana Carla Bridi, Osnir Claudiano da Silva, Carlos Roberto Lyra da Silva, Thiago Quinellato Louro, Daniel Aragão Machado

**Affiliations:** I Universidade Federal do Estado do Rio de Janeiro. Rio de Janeiro, Rio de Janeiro, Brazil; II Universidade do Estado do Rio de Janeiro. Rio de Janeiro, Rio de Janeiro, Brazil; III Universidade Federal Fluminense. Rio das Ostras, Rio de Janeiro, Brazil

**Keywords:** Perioperative Nursing, Nursing Care, Perioperative Care, Robotic Surgical Procedures, Validation Study, Enfermagem Perioperatória, Cuidados de Enfermagem, Assistência Perioperatória, Procedimentos Cirúrgicos Robóticos, Estudo de Validação, Enfermagem Perioperatória, Cuidados de Enfermagem, Assistência Perioperatória, Procedimentos Cirúrgicos Robóticos, Estudo de Validação

## Abstract

**Objective:**

To develop and validate an instrument to assist in the systematization of perioperative nursing care in robotic surgery.

**Methods:**

Methodological study developed in four phases: content survey; textual elaboration; content validation by the group of expert judges and target audience; and elaboration of the electronic instrument layout.

**Results:**

Eleven expert judges and seven evaluators of the target audience participated. For validation, the Content Validity Index (CVI) was used with a 0.78 cutoff point. The instrument total CVI after evaluation was 0.90 by the expert judges and 0.88 by the target audience.

**Conclusion:**

The tool built was proved satisfactory for the systematization of perioperative nursing care. The instrument construction was based on the updated scientific literature and validated by the expert judges and target audience.

## RESUMO

**Objetivo:**

Desenvolver e validar um instrumento para auxiliar na sistematização da assistência de enfermagem perioperatória em cirurgia robótica.

**Métodos:**

Estudo metodológico desenvolvido em quatro fases: levantamento do conteúdo; elaboração textual; validação do conteúdo pelo grupo de juízes especialistas e público-alvo; e elaboração do layout do instrumento eletrônico.

**Resultados:**

Participaram 11 juízes especialistas e 7 avaliadores do público-alvo. Para validação, utilizou-se o Índice de Validade de Conteúdo (IVC) com ponto de corte em 0,78. O IVC total do instrumento após avaliação foi de 0,90 pelos juízes especialistas e 0,88 pelo público-alvo.

**Conclusão:**

A ferramenta construída se mostrou satisfatória para realização da sistematização da assistência de enfermagem perioperatória. A construção do instrumento foi embasada na literatura científica atualizada e validada pelos juízes especialistas e público-alvo.

## RESUMEN

**Objetivo:**

Desarrollar y validar un instrumento para auxiliar en la sistematización de la atención de enfermería perioperatoria en cirugía robotizada.

**Métodos:**

Estudio metodológico desarrollado en cuatro fases: análisis del contenido; elaboración textual; validación del contenido por el equipo de jueces especialistas y público objetivo; y elaboración del diseño del instrumento electrónico.

**Resultados:**

Participaron 11 jueces especialistas y 7 evaluadores del público objetivo. Para validación, se utilizó el Índice de Validez de Contenido (IVC) con punto de corte en 0,78. El IVC total del instrumento después de la evaluación fue de 0,90 por los jueces especialistas y 0,88 por el público objetivo.

**Conclusión:**

La herramienta construida se mostró satisfactoria para realización de la sistematización de la atención de enfermería perioperatoria. La construcción del instrumento fue basada en la literatura científica actualizada y validada por los jueces especialistas y público objetivo.

## INTRODUCTION

The use of technological innovations in health care has increased significantly in recent years, especially regarding surgical interventions. The surgical modality highlighted is robotic surgery, which aims to provide the benefits of this minimally invasive technique, combined with the lower risk of complications^(1, 2, 3, 4)^.

Robotic surgery is defined as a minimally invasive, high-precision technology that, through three-dimensional imaging, allows the surgeon to perform more accurate procedures even away from the patient through the console, reducing interference of the surgeon’s hands and instrumentation mobility, especially in operating fields of restricted spaces^(4, 5)^.

In Brazil, robotic technology arrived in 2008 in São Paulo. Its incorporation into the routine of any hospital requires adjustments, from structural changes in the operating room and purchase of equipment to professionals trained and qualified to manipulate the robot^(3, 4, 5)^.

Nurses’ participation is essential for the accomplishment of this surgical modality, acting in all perioperative period phases, especially the intraoperative. These professional acts proactively in the robotic system planning and in the provision of inputs and equipment needed for the medical specialty, without forgetting patient safety and care procedures such as care involving surgical positioning^(3, 6, 7)^.

In addition, some challenges are faced in this scenario, such as development of new skills, formation of a qualified and specialized nursing team in the face of technological innovations in the field of robotics, and managerial and care attributions pertinent to the perioperative nurse, such as intraoperative care and minimization of risks and complications related to the procedure^(1, 3, 6, 7)^.

In this context, nurses need to structure nursing care to provide safety and quality of care. The methodological tool that enables this is the systematization of perioperative nursing care (SPNC). Its application organizes and systematizes the practice with a scientific basis in an individualized way. However, despite the benefits, the literature indicates that some services still face difficulty in performing this care completely^(1, 8, 9, 10)^.

We understand that the role of nurses in assisting patients undergoing robotic surgery is recent, but of paramount importance, because it is presented as a new field of action; and, as the direct role of nurses is required in the three phases of the perioperative period, this surgical procedure becomes ideal for performing SPNC in an integral way^(1, 3, 11)^.

Thus, the question is: “Would the development of a validated instrument facilitate the application of SPNC aimed at patients undergoing robotic surgery?”.

## OBJECTIVE

To develop and validate an instrument to assist in SPNC in robotic surgery.

## METHODS

### Ethical aspects

The study was submitted to and approved by the Research Ethics Committee (REC) of the Federal University of the State of Rio de Janeiro (UNIRIO) and the Marcílio Dias Naval Hospital (HNMD), with an opinion attached to the manuscript submission. Resolution No.466/12, which deals with guidelines and regulatory standards for research involving human beings, and Resolution No. 510/16, as research conducted in an online environment, in accordance with national ethics guidelines were respected^(12, 13)^.

Informed Consent was obtained from all individuals involved in the study online before the start of data collection.

### Study design, period and place

This is a methodological study that aims to build and validate a care instrument seeking to improve research or practice. It was developed in four stages: 1) literature review to survey the content; 2) textual elaboration; 3) content validation by the group of expert judges and target audience; 4) completion of instrument after content validation^(14)^.

SQUIRE 2.0 guidelines were followed to improve quality standards and methodological guidelines for publications^(15)^.

The study development happened between February 2022 and August 2022.

### Population or sample; inclusion and exclusion criteria

The instrument created underwent two groups of evaluators. The first one regards specialists in the field of surgical center and/or nursing assistance to robotic surgery and who had experience in the application of SPNC in that environment. For this group, the inclusion criteria established were: graduation in Nursing; specialization or master’s degree or doctorate in surgical center or robotic surgery; and experience in the area.

The second group, formed by nurses of the service (target audience), was also invited to evaluate the instrument. To compose this group, the following inclusion criteria were used: graduation in Nursing; experience in the surgical center of the hospital chosen as the study scenario; and a minimum of three months of experience in the sector.

### Study protocol

In the study’s first and second stages, there was a literature review to support the instrument elaboration.

In the third stage, the following classification was considered in two groups: 1) content judges/expert judges (professionals with expertise in the topic addressed); and 2) target audience (nurses of the service). It was important, for the validation of the content, that the judges were qualified to evaluate the relevance and representativeness of the content to compose the technology^(2, 16, 17, 18)^.

The scientific literature recommends a minimum number of five experts; thus, 11 expert judges and 7 evaluators of the target audience participated^(2, 16, 17)^.

For the recruitment of experts, the method used was intentional sampling. The data collection kit was sent to the study groups of the area at universities (by e-mail) and groups of surgical center professionals (by WhatsApp), consisting of an invitation letter and access link of the instrument via Google Forms, in which the ICF, the textual base elaborated (SPNC instrument) and the form for content validation were attached^(14)^.

The judges had 20 calendar days to respond to the form via access link. The estimated filling time was 10 to 20 minutes. A new contact was made with those who did not respect the deadline, clarifying the importance of participation and evaluation, as well as granting an additional five days. After this deadline, the form in Google Forms was closed.

### Analysis of results and statistics

For data collection, an instrument was prepared via Google Forms divided into two parts: Part I, with data regarding characterization and professional experience of the judges; and Part II, containing the instructions for completing the instrument and the evaluation items for content validation.

The collected data were stored and organized in Microsoft Excel, version 11.0. Descriptive statistics were used to analyze the social and professional variables of the expert judges and the target audience, based on the literature relevant to the subject.

Part II of the instrument was elaborated with questions on the content evaluation regarding comprehension, coherence of information, language, presentation and layout, ease of handling, with items distributed in three blocks and a field for general comments and suggestions^(2, 17, 19)^.

Block 1 concerns the Objectives, with seven items referring to the purposes, goals or ends that are desired to be achieved with the use of technology. Block 2 is about Structure and Presentation, with eight items regarding how to present the information in the instrument. This includes its overall organization, structure, presentation strategy, coherence, and formatting. Finally, Block 3 refers to relevance, with three items related to the characteristics that assess the degree of significance of the care material presented^(2, 17, 19)^.

The validation instrument used the Likert scale, which employs a classification technique consisting of several statements (items) that express points of view on a topic. For each statement, the following degrees of valuation were considered: 1 = Inadequate; 2 = Partially Adequate; 3 = Adequate; 4 = Totally Adequate.

For the instrument to be validated, it must have a Content Validity Index (CVI) greater than or equal to 0.78. The CVI measures the proportion of agreement on the items evaluated in the instrument. The CVI is calculated by summing the scores of items evaluated as 3 (Adequate) and 4 (Totally Adequate) divided by the total number of responses^(2, 14, 19)^.

The content was validated for each item belonging to each block in isolation, so that the CVI of each isolated block was then calculated and, finally, the total CVI for the instrument as a whole.

## RESULTS

**Figure 1:**
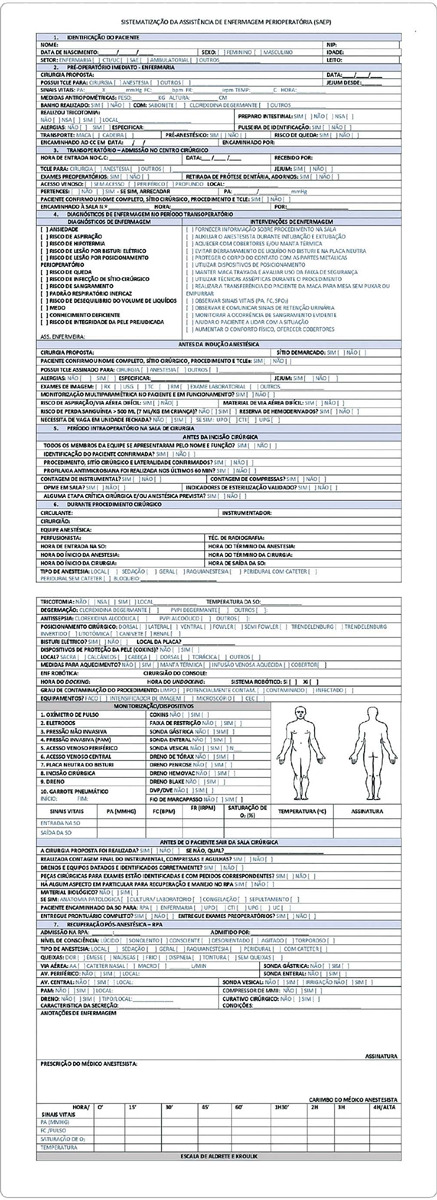
Systematization of Nursing Care Instrument (SPNC) - Part 1

**Figure 2:**
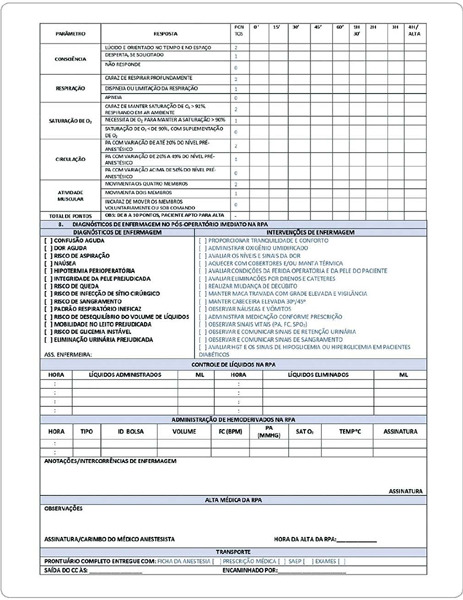
Systematization of Nursing Care Instrument (SPNC) - Part 2

During the textual elaboration of the instrument (Figures 1 and 2) to be validated, other studies sought relevant information on patient identification, safe surgery checklist, care performed preoperatively, intraoperatively, and postoperatively with the respective nursing diagnoses and interventions. The purpose is to deliver an updated instrument based on the scientific literature, enabling the registration of nursing care to be more complete and comprehensive.

### Characterization of expert judges and target audience

The study population was divided into expert judges and target audience (nurses of the service). In both groups, the response rate was 100%. All participants were female. Table 1 shows the characterization of expert judges and Table 2 shows the characterization of target audience.

**Table 1:** Socioeconomic profile of expert judges. Rio de Janeiro, Rio de Janeiro, Brazil, 2022

Variables	Expert judges	
	f	%
Gender		
Female	11	100
Age group (years)		
31–40	7	63.6
41–50	1	9.1
51-60	2	18.2
61 or more	1	9.1
Degree		
Specialization	6	54.5
Master's Degree	2	18.2
Doctorate	3	27.3
Length of professional experience (years)		
0-5	1	9.1
5-10	1	9.1
10-20	5	45.4
20-30	1	9.1
More than 30	3	27.3
Are you specialized in surgical center and/or robotic surgery?		
Yes	9	81.8
No	2	18.2
Work area		
Care	3	27.3
Managerial	4	36.4
Teaching and/or research	3	27.3
Sales	1	9.0
Institution where you work		
Public	7	63.6
Private	4	36.4
Previous experience with instrument construction and/or validation?		
Yes	9	81.8
No	2	18.2

*f - absolute frequency; % - percentage*

**Table 2:** Socioeconomic profile of the target audience, Rio de Janeiro, Rio de Janeiro, Brazil, 2022

Variables	Target audience	
	n	%
Gender		
Female	7	100
Age group (years)		
31–40	4	57.2
41 – 50	3	42.8
Degree		
Specialization	6	85.7
Master's Degree	1	14.3
Length of professional experience (years)		
5-10	4	57.2
10-20	2	28.5
20-30	1	14.3
Are you specialized in surgical center and/or robotic surgery?		
Yes	5	71.5
No	2	28.5
Work area		
Care	6	85.7
Managerial	1	14.3
Institution where you work		
Public	7	100
Private	-	
Previous experience with instrument validation?		
Yes	1	14.3
No	6	85.7

*f - absolute frequency; % - percentage*

When analyzing the data, we can emphasize that the prevalent age in both groups was between 31 and 40 years old, with 63.6% of the judges and 57.2% of the target audience. Regarding the degree, a similar behavior was observed between the groups, in which the specialization prevailed for the majority, followed by the doctorate only among the judges.

Regarding the specialization in surgical center and/or robotic surgery, the two groups presented more than 70% prevalence, which demonstrates experience in the area and brings a richer look at the theme, with more relevant contributions to the instrument evaluation.

Previous participation in studies on instrument validation showed percentage differences between groups. Most of the (81.8%) expert judges had previously worked with this type of approach, while among the target audience, only one professional (14.3%) had previously worked with it.

The category of expert judges was expected to present the highest percentage in this item so that a more robust evaluation could be made based on the experience in other previous studies. The lack of familiarity observed in the target audience group with the methodology used could bring a little more difficulty for some people, but following the instructions left on the instrument, their participation would be possible.

### Validation of instrument content by expert judges and target audience

The Content Validity Index (CVI) was used to validate the instrument, with a cutoff point of 0.78. To calculate the CVI, three approaches were adopted: first, the calculation of content for each item belonging to each block alone (CVIi), considering the number of judges who evaluated the item as “totally adequate” and “adequate”; second, the calculation of content for each isolated block (CVIb) was performed; finally, for the third approach, the instrument as a whole was evaluated by the average proportion of the items evaluated as “totally adequate” and “adequate” by the number of evaluators (CVIt)^(2, 13, 14, 17)^.

It is noteworthy that the items evaluated with grade “2” (partially adequate) or “1” (inadequate) were analyzed and corrected.

Initially, the expert judges and the target audience evaluated Block 1, which referred to the objective of the instrument, with regard to the purposes, goals or ends to be achieved with the use of technology (seven items) — data presented in Table 3.

**Table 3:** Content validation by expert judges and target audience, Rio de Janeiro, Rio de Janeiro, Brazil, 2022

Evaluated items	CVI	
	Expert judges	Target audience
1.1 Block 1: Objectives	**0.89**	**0.91**
1.1 The text is compatible with the target audience, including SPNC.	0.9	0.85
1.2 The content covered is adequate for performing SPNC in the perioperative period of robotic surgery.	0.9	0.85
1.3 Guidelines presented are necessary and have been addressed correctly.	0.9	1
1.4 It causes change in behavior and attitudes.	0.9	1
1.5 Information is updated.	0.81	1
1.6 The content meets the work proposal.	0.9	0.72
1.7 It can be applied in practice.	0.9	1
2.2 Block 2: Structure and Presentation	**0.87**	**0.83**
2.1 Application-type technology is appropriate to assist in SPNC in the perioperative period of robotic surgery.	1	0.83
2.2 The language is appropriate for the target audience.	0.9	1
2.3 The information is presented clearly and objectively.	0.9	1
2.4 The instrument has adequate size, that is, it is not tiring.	0.6	0.42
2.5 The formatting is adequate (letter, size, space).	0.9	0.85
2.6 The size and color of the letters of the headings, subheadings and text are adequate.	0.9	0.66
2.7 The writing style corresponds to the level of knowledge of the target audience.	0.9	1
2.8 There is a logical sequence of proposed content.	0.9	0.85
3.3 Block 3: Relevance	**1**	**0.95**
3.1 The material includes the matters necessary to carry out the SPNC.	1	0.85
3.2 Is the instrument adequate for use by any nurse with experience in the operating theatre and/or robotic surgery?	1	1
3.3 Does the instrument contemplate and integrate the main points of patient care in the perioperative period?	1	1
Total CVI of the instrument	**0.9**	**0.88**

*CVI - Content Validity Index*

As for the objective/purpose of the instrument (Block 1), it was considered validated, since, when evaluated in isolation, the CVIi ranged from 0.81 to 1.0 among the evaluators; and, when the entire block was evaluated, it reached CVIb1 of 0.89 by the expert judges and 0.93 by the target audience, values well above the established cutoff point.

Then, Block 2 was evaluated, which deals with the structure and presentation, that is, how to present the information in the instrument. This includes its overall organization, structure, presentation strategy, coherence, and formatting (eight items) — data presented in Table 3. In this regard, the instrument was considered validated, since, when the entire block was evaluated, it reached a CVIb1 of 0.87 by the expert judges and 0.83 by the target audience, values above the established cutoff point. However, when evaluated in isolation, the CVIi ranged from 0.42 to 1.0 among the evaluators.

Item 2.4, “The instrument has adequate size, that is, it is not tiring”, has the CVIi of 0.60 among the expert judges and 0.42 among the target audience. However, the instrument proposed for evaluation includes the junction of three sheets of institutional forms that needed to be updated and adapted to the standards suggested in the literature, following the recommendations of good practices. Nevertheless, after evaluation, it was possible to further reduce its extension, maintaining the current character.

Item 2.6, “The size and color of the letters of the headings, subheadings and text is adequate”, had the CVIi among the target audience of 0.66, being revised and corrected. Although these two items recorded CVIi in isolation below the established cutoff point, this did not impact the evaluation of Block 2 in full, which was validated.

It was concluded with Block 3, which seeks to evaluate the relevance of the instrument, referring to the characteristics that measure the degree of significance of the care material presented (three items) — data presented in Table 3.

Regarding relevance, the instrument was validated, having presented CVIi ranging between 0.85 and 1.0 by the evaluators; and, when the entire block was evaluated, it presented CVIb3 of 1.0 by the expert judges and 0.95 by the target audience.

Finally, the CVIt of the full instrument was calculated (Table 3), which was 0.90 by the expert judges and 0.89 by the target audience, being considered an instrument validated by both groups of evaluators.

## DISCUSSION

For the construction of the instrument, recent studies on the subject were sought in the literature, with proposals for evaluative items to build a tool that will assist in perioperative care, making it more integral, individualized, and safe.

SPNC is a methodological tool recommended by the regulatory bodies of the profession, with its mandatory implementation in health institutions. In addition, it is a nurse’s private activity, but it must count on the participation of other professionals of the nursing team in all process stages. This tool not only organizes care by conferring safety, integrality, and individuality, but also has legal value because it is the documentation of professional practice for the purposes of process audits, civil responsibilities, and continuing education^(1, 8, 20, 21, 22)^.

Studies in the area suggest that the lack of registration of nursing care makes the work developed by the team invisible, in addition to raising doubts about whether the care was performed, which may call into question the quality of care provided. This quality is directly related to the good anesthetic-surgical outcome of patients, and a supported practice is of paramount importance^(1, 8, 20, 21, 22)^.

In this context, we sought to include, in the elaboration of the instrument, information relevant to the surgical safety checklist, such as patient identification, presence of consent terms, name of the procedure, surgical team, among other information necessary for the provision of care throughout the period^(8, 11, 20, 21, 22)^.

Then, we sought to correlate this information with the possible nursing diagnoses (ND) that contemplated the three phases of the perioperative period. However, there were few studies that dealt with the ND in the perioperative period and/or that were associated with the occurrence of robotic surgery. Thus, the validated instrument is expected to assist nurses in structuring care with the identification of possible risks and choosing the most appropriate interventions^(8, 11, 20, 21, 22)^.

The validation step of the proposed instrument was carried out carefully and in detail. Such conduct was observed in other validation studies both in the construction phase of the instrument and in the recruitment stage of expert judges. It is essential that professionals of notorious knowledge on the subject participate in the content evaluation, so they can contribute consistently in the construction of the tool, adding greater scientific rigor and reaching the proposed objective^(2, 18, 23, 24)^.

One of the difficulties encountered in the content elaboration, pointed by the judges during the instrument validation, was to compile all the pertinent information of the assistance for the adequate registration of the actions without leaving the instrument extensive. It was one of the items with a low CVI index in the opinion of evaluators, but it did not compromise the instrument validation.

Few suggestions were made by the expert judges. They were carefully evaluated and analyzed according to scientific studies and good recommended practices on the subject, and modifications were made when necessary.

The proposed instrument was also validated by nurses of the service – the target audience – so that it could be inserted in the routine of the service in the best possible way. Thus, we believe it is possible to awaken the gaze of professionals involved in robotic surgery to the benefits that the application of SPNC in full would bring to care practice^(24, 25)^.

### Study limitations

One of the study limitations was to identify: a timid movement of Brazilian nursing in the publication of national articles that addressed the validation of an instrument for SPNC; and the need for more research on nursing diagnoses in the perioperative period and in robotic surgery.

### Contributions to Nursing and Health

The study contributes to the practice of nurses working in perioperative care, highlighting the importance of nursing focusing on SPNC, understanding it as a tool that brings technical-scientific support to care practice. In this sense, it is necessary to develop tools that will assist in direct patient care of surgical patients undergoing robotic surgery.

## CONCLUSIONS

The study objectives were achieved, with the creation of an instrument based on an extensive literature review and on the experiences by the evaluators and the researcher.

The proposed instrument was validated and proved to be perfectly applicable to assist the implementation of SPNC in the context proposed in the study, with the objective of providing individualized, comprehensive, quality and safety care. In addition, we observed that nurses, in the surgical care scenario, have a mediating role among other professionals to guide the actions and care provided to patients in the perioperative period, a time of extreme vulnerability.

The importance of nurses to stay well-informed on nursing processes, systematization of care and innovative technologies such as robotic surgery is highlighted in order to promote quality and safe care to patients.

## Supplementary Material

0034-7167-reben-76-S4-e20220666-suppl01
