# Supplementary material for: Systematization of perioperative nursing care in robotic surgery: instrument validation
Source: Rev Bras Enferm. 2023 Dec 8;76(Suppl 4):e20220666. doi: 10.1590/0034-7167-2022-0666 (PMC10704656; doi:10.1590/0034-7167-2022-0666)
Supplement: 0034-7167-reben-76-S4-e20220666-suppl01 [file 0034-7167-reben-76-s4-e20220666-suppl01.pdf]

SISTEMATIZAÇAO DA ASSISTÊNCIA DE ENFERMAGEM PERIOPERATÓRIA (SAEP)

1. IDENTIFICAÇÃO DO PACIENTE

**NOME:**	**NIP:**
**DATA DE NASCIMENTO:**______/______/_____ **SEXO:** [ ] FEMININO [ ] MASCULINO	**IDADE:**
**SETOR:** ENFERMARIA [ ] CTI/UC [ ] SAE [ ] AMBULATORIAL [ ] OUTROS_______________	**LEITO:**

2. PRÉ-OPERATÓRIO IMEDIATO - ENFERMARIA

**CIRURGIA PROPOSTA:**	**DATA:**____/____/____
**POSSUI TCLE PARA:** CIRURGIA [ ] ANESTESIA [ ] OUTROS [ ] _____________________	**JEJUM DESDE:**_______

SINAIS VITAIS: PA:______X_______mmhg FC:_______bpm FR:__________irpm TEMP:_________C HORA:___________

MEDIDAS ANTROPOMÉTRICAS: PESO:_________KG ALTURA: _________ CM

BANHO REALIZADO: SIM [ ] NÃO [ ] **COM:** SABONETE [ ] CLOREXIDINA DEGERMANTE [ ] OUTROS____________

**REALIZOU TRICOTOMIA:** NÃO [ ] NSA [ ] SIM [ ] LOCAL___________________________	**PREPARO INTESTINAL:** SIM [ ] NÃO [ ] NSA [ ]
**ALERGIAS:** NÃO [ ] SIM [ ] **ESPECIFICAR:**________________	**PULSEIRA DE IDENTIFICAÇÃO:** SIM [ ] NÃO [ ]

**TRANSPORTE:** MACA [ ] CADEIRA [ ]	**PRÉ-ANESTÉSICO:** SIM [ ] NÃO [ ]	**RISCO DE QUEDA:** SIM [ ] NÃO [ ]

**ENCAMINHADO AO CC EM DATA: / /**	**ENCAMINHADO POR:**

3. TRANSOPERATÓRIO – ADMISSÃO NO CENTRO CIRÚRGICO

**HORA DE ENTRADA NO C.C:** ____________	**DATA:**___ /____ /____	**RECEBIDO POR:**
**TCLE PARA:** CIRURGIA [ ] ANESTESIA [ ] OUTROS [ ] ________________		**JEJUM:** SIM [ ] NÃO [ ]

**EXAMES PREOPERATÓRIOS:** SIM [ ] NÃO [ ]	**RETIRADA DE PRÓTESE DENTÁRIA, ADORNOS:** SIM [ ] NÃO [ ]

ACESSO VENOSO: [ ] SEM ACESSO [ ] PERIFERICO [ ] PROFUNDO **LOCAL:** __________________

**PERTENCES:** [ ] NÃO [ ] SIM **- SE SIM, ARRECADAR**	**PA:** ________/___________MMHG

PACIENTE CONFIRMOU NOME COMPLETO, SÍTIO CIRÚRGICO, PROCEDIMENTO E TCLEs: SIM [ ] NÃO [ ]

**ENCAMINHADO A SALA** N.º ______________ **HORA:**______________	**POR:**_____________________

4. DIAGNÓSTICOS DE ENFERMAGEM NO PERÍODO TRANSOPERATÓRIO

DIAGNÓSTICOS DE ENFERMAGEM	INTERVENÇÕES DE ENFERMAGEM
[ ] ANSIEDADE<br>[ ] RISCO DE ASPIRAÇAO<br>[ ] RISCO DE HIPOTERMIA<br>[ ] RISCO DE LESÃO POR BISTURI ELETRICO<br>[ ] RISCO DE LESÃO POR POSICIONAMENTO PERIOPERATÓRIO<br>[ ] RISCO DE QUEDA<br>[ ] RISCO DE INFECÇÃO DE SITIO CIRÚRGICO<br>[ ] RISCO DE SANGRAMENTO<br>[ ] PADRÃO RESPIRATÓRIO INEFICAZ<br>[ ] RISCO DE DESEQUILIBRIO DO VOLUME DE LIQUÍDOS<br>[ ] MEDO<br>[ ]CONHECIMENTO DEFICIENTE<br>[ ] RISCO DE INTEGRIDADE DA PELE PREJUDICADA<br><br>ASS OF ENFERMEIRA:	[ ] FORNECER INFORMAÇÃO SOBRE PROCEDIMENTO NA SO<br>[ ] AUXILIAR O ANESTESISTA DURANTE INTUBAÇÃO E EXTUBAÇÃO<br>[ ] AQUECER COM COBERTORES, E /OU MANTA TÉRMICA<br>[ ] EVITAR DERRMAMENTO DE LIQUÍDO NO BISTURI E NA PLACA NEUTRA<br>[ ] PROTEGER O CORPO DO CONTATO COM AS PARTES METÁLICAS<br>[ ] UTILIZAR DISPOSITIVOS DE POSICIONAMENTO<br>[ ] MANTER MACA TRAVADA E AVALIAR USO DA FAIXA DE SEGURANÇA<br>[ ] UTILIZAR TÉCNICAS ASSÉPTICAS DURANTE O PROCEDIMENTO<br>[ ] REALIZAR A TRANSFERÊNCIA DO PACIENTE DA MACA PARA MESA SEM PUXAR OU EMPURRAR<br>[ ] OBSERVAR SINAIS VITAIS (PA, FC, SPO2)<br>[ ] OBSERVAR E COMUNICAR SINAIS DE RENTENÇÃO URINÁRIA<br>[ ] MONITORAR A OCORRÊNCIA DE SANGRAMENTO EVIDENTE<br>[ ] AJUDAR O PACIENTE A LIDAR COM A SITUAÇÃO<br>[ ] AUMENTAR O CONFORTO FÍSICO, OFERECER COBERTORES

ANTES DA INDUÇÃO ANESTÉSICA

**CIRURGIA PROPOSTA:**	**SÍTIO DEMARCADO:** SIM [ ] NÃO [ ]

PACIENTE CONFIRMOU NOME COMPLETO, SÍTIO CIRÚRGICO, PROCEDIMENTO E TCLEs: SIM [ ] NÃO [ ]

POSSUI TCLE ASSINADO PARA: CIRURGIA [ ] ANESTESIA [ ] OUTROS [ ] ___________________

**ALERGIAS:** NÃO [ ] SIM [ ] **ESPECIFICAR:**____________________	**JEJUM:** SIM [ ] NÃO [ ]

EXAMES DE IMAGEM: [ ] RX [ ] USG [ ] TC [ ] RM [ ] EXAME LABORATORIAL [ ] OUTROS_______________

MONITORIZAÇAO MULTIPARAMETRICA NO PACIENTE E EM FUNCIONAMENTO? SIM [ ] NÃO [ ]

**RISCO DE ASPIRAÇÃO/ VIA AÉREA DIFÍCIL:** SIM [ ] NÃO[ ]	**MATERIAL DE VIA AÉREA DIFICIL:** SIM [ ] NÃO [ ]

RISCO DE PERDA SANGUÍNEA > 500ML (7ML/KG EM CRIANÇA)? NÃO [ ] SIM [ ] **RESERVA DE HEMODERIVADOS?** SIM [ ] NÃO [ ]

NECESSITA DE VAGA DE UNIDADE FECHADA? NÃO [ ] SIM [ ] SE SIM, UPO [ ] CTI [ ] UPG [ ]

5. PERÍODO INTRAOPERATÓRIO NA SALA DE CIRURGIA

ANTES DA INCISÃO CIRÚRGICA

TODOS OS MEMBROS DA EQUIPE SE APRESENTARAM PELO NOME E FUNÇÃO? SIM [ ] NÃO [ ]

IDENTIFICAÇÃO DO PACIENTE CONFIRMADA? SIM [ ] NÃO [ ]

PROCEDIMENTO, SITÍO CIRÚRGICO E LATERALIDADE CONFIRMADAS? SIM [ ] NÃO [ ]

PROFILAXIA ANTIMICROBIANA FOI REALIZADA NOS ÚLTIMOS 60 MIN? SIM [ ] NÃO [ ]

**CONTAGEM DE INSTRUMENTAL?** SIM [ ] NÃO [ ]	**CONTAGEM DE COMPRESSAS?** SIM [ ] NÃO [ ]
**OPME EM SALA?** SIM [ ] NÃO [ ]	**INDICADORES DE ESTERILIZAÇÃO VALIDADO?** SIM [ ] NÃO [ ]

ALGUMA ETAPA CRÍTICA CIRÚRGICA E/OU ANESTÉSICA PREVISTA? SIM [ ] NÃO [ ]

6. DURANTE PROCEDIMENTO CIRÚRGICO

CIRCULANTE:	INSTRUMENTADOR:
CIRURGIÃO:	
EQUIPE ANESTÉSICA:	
PERFUSIONISTA:	TÉC. DE RAIOX:
HORA DE ENTRADA NA SO:	HORA DO TÉRMINO DA ANESTESIA:
HORA DO ÍNICIO DA ANESTSIA:	HORA DO TÉRMINO DA CIRURGIA:
HORA DO ÍNICIO DA CIRURGIA:	HORA DE SAÍDA DA SO:

TIPO DE ANESTESIA: LOCAL [ ] SEDAÇÃO [ ] GERAL [ ] RAQUIANESTESIA [ ] PERIDURAL COM CATETER [ ] PERIDURAL SEM CATETER [ ] BLOQUEIO:___________________________

TRICOTOMIA: NÃO [ ] NSA [ ] SIM [ ] LOCAL________________ TEMPERATURA DA SO:_______________

DEGERMAÇÃO: CLOREXIDINA DEGERMANTE [ ] PVPI DEGERMANTE [ ] OUTROS [ ]:

ANTISSEPISIA: CLOREXIDINA ALCOÓLICA [ ] PVPI ALCOÓLICO [ ] OUTROS [ ]:

POSICIONAMENTO CIRÚRGICO: DORSAL [ ] LATERAL __ [ ] VENTRAL [ ] FOWLER [ ] SEMI FOWLER [ ] TRENDELENBURG [ ] TRENDELENBURG INVERTIDO [ ] LITOTÔMICA [ ] CANIVETE [ ] RENAL [ ]

BISTURI ELÉTRICO? SIM [ ] NÃO [ ] LOCAL DA PLACA? ___________________

DISPOSITIVOS DE PROTEÇÃO DA PELE (COXINS)? NÃO [ ] SIM [ ]
LOCAL? SACRA [ ] CALCÂNEOS[ ] CABEÇA[ ] DORSAL[ ] TORÁCICA [ ] OUTROS[ ]

MEDIDAS PARA AQUECIMENTO? NÃO [ ] SIM[ ] MANTA TÉRMICA [ ] INFUSÃO VENOSA AQUECIDA [ ] COBERTOR[ ]

ENF ROBÓTICA: CIRURGIÃO DO CONSOLE:

HORA DO DOCKING: HORA DO UNDOCKING: SISTEMA ROBÓTICO: Si [ ] Xi[ ]

GRAU DE CONTAMINAÇÃO DO PROCEDIMENTO: LIMPO [ ] POTENCIALMENTE CONTAM. [ ] CONTAMINADO [ ] INFECTADO [ ]

EQUIPAMENTOS? FACO [ ] INTENSIFICADOR DE IMAGEM [ ] MICROSCÓPIO [ ] CEC [ ]

MONITORIZAÇÃO/DISPOSITIVOS	
1. OXIMETRO DE PULSO	COXINS NÃO [ ] SIM [ ]
2. ELETRODOS	FAIXA DE RESTRIÇÃO NÃO [ ] SIM [ ]
3. PRESSÃO NÃO INVASIVA	SONDA GÁSTRICA NÃO [ ] SIM [ ]
4. PRESSÃO INVASIVA (PAM)	SONDA ENTERAL NÃO [ ] SIM [ ]
5. ACESSO VENOSO PERIFÉRICO	SONDA VESICAL NÃO [ ] SIM [ ] N___
6. ACESSO VENOSO CENTRAL	DRENO DE TÓRAX NÃO [ ] SIM [ ]
7. PLACA NEURTA DO BISTURI	DRENO PENROSE NÃO [ ] SIM [ ]
8. INCISÃO CIRÚRGICA	DRENO HEMOVAC NÃO [ ] SIM [ ]
9. DRENO	DRENO BLAKE NÃO [ ] SIM [ ]
10. GARROTE PENEUMÁTICO	DVP / DVE NÃO [ ] SIM [ ]
INÍCIO: FIM:	FIO DE MARCAPASSO NÃO [ ] SIM [ ]

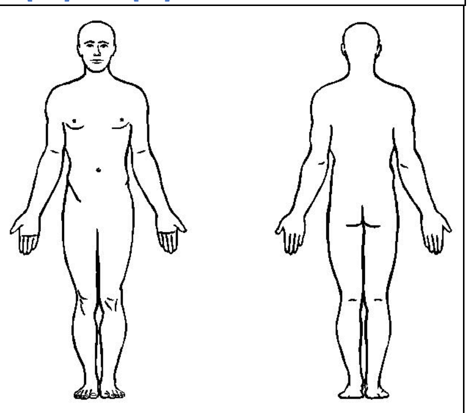

SINAIS VITAIS	PA (MMHG)	FC (BPM)	FR (IRPM)	SATURAÇÃO DE O2 (%)	TEMPERATURA (°C)	ASSINATURA
ENTRADA NA SO						
SAÍDA DA SO						

ANTES DO PACIENTE SAIR DA SALA CIRÚRGICA

A CIRURGIA PROPOSTA FOI REALIZADA? SIM [ ] NÃO [ ] SE, NÃO QUAL?_____________________________________

REALIZADA CONTAGEM FINAL DE INSTRUMENTAL, COMPRESSA E AGULHAS? SIM [ ] NÃO [ ]

DRENOS E EQUIPOS DATADOS E IDENTIFICADOS CORRETAMENTE? SIM [ ] NÃO [ ]

PEÇAS CIRURGICA PARA EXAMES ESTÃO IDENTIFICADAS E COMPEDIDOS CORRESPONDENTES? SIM [ ] NÃO [ ]

HÁ ALGUM ASPECTO EM PARTICULAR PARA RECUPERAÇÃO E MANEJO NO RPA SIM [ ] NÃO [ ]

MATERIAL BIOLÓGICO? NÃO [ ] SIM [ ]
SE SIM: ANATOMIA PATOLOGICA [ ] CULTURA/ LABORATÓRIO [ ] CONGELAMENTO [ ] SEPULTAMENTO [ ]

PACIENTE ENCAMINHADO DA SO PARA: RPA [ ] ENFERMARIA [ ] UPO [ ] CTI [ ] UPG [ ] UC [ ]

ENTREGUE PRONTUÁRIO COMPLETO? SIM [ ] NÃO [ ] ENTREGUE EXAMES PREOPERATÓRIOS? SIM [ ] NÃO [ ]

7. RECUPERAÇÃO PÓS ANESTÉSICA – RPA

ADMISSÃO NA RPA: ________:__________ ADMITIDO POR:_____________________

NÍVEL DE CONSCIÊNCIA: LÚCIDO [ ] SONOLENTO [ ] CONSCIENTE [ ] DESORIENTADO [ ] AGITADO [ ] TORPOROSO [ ]

TIPO DE ANESTESIA: LOCAL [ ] SEDAÇÃO [ ] GERAL [ ] RAQUIANESTESIA [ ] PERIDURAL [ ] COM CATETER[ ]

QUEIXAS: DOR [ ] ÊMESE [ ] NAÚSEAS [ ] FRIO [ ] DISPNEIA [ ] TONTURA [ ] SEM QUEIXAS [ ]

VIA AÉREA: AA [ ] CATÉTER NASAL [ ] MACRO [ ] _________ L/MIN	SONDA GÁSTRICA: NÃO [ ] SIM [ ]
AV. PERIFÉRICO: NÃO [ ] SIM [ ] LOCAL:	SONDA ENTERAL: NÃO [ ] SIM [ ]
AV. CENTRAL: NÃO [ ] SIM [ ] LOCAL:	SONDA VESICAL: NÃO [ ] SIM [ ] IRRIGAÇÃO NÃO [ ] SIM [ ]
PAM: NÃO [ ] SIM [ ] LOCAL:	COMPRESSOR DE MMII: NÃO [ ] SIM [ ]
DRENO: NÃO [ ] SIM [ ] TIPO / LOCAL:_______________	CURATIVO CIRÚRGICO: NÃO [ ] SIM [ ]

CARACTERISTICA DA SECREÇÃO:________________________	CONDIÇÕES:____

ANOTAÇÕES DE ENFERMAGEM

ASSINATURA

PRESCRIÇÃO MÉDICO ANESTESISTA:

CARIMBO ANESTESISTA

HORA/ SINAIS VITAIS	O’	15’	30’	45’	60’	1H30’	2H	3H	4H/ ALTA
PA (MMHG)									
FC /PULSO									
SATURAÇÃO DE O2									
TEMPERATURA									

ESCALA DE ALDRETE E KROULIK

PARÂMETRO	RESPOSTA	PONTOS	0 ‘	15’	30’	45’	60’	1H 30’	2H	3H	4H / ALTA
CONSCIÊNCIA	LÚCIDO E ORIENTADO NO TEMPO E NO ESPAÇO	2									
	DESPERTA, SE SOLICITADO	1									
	NÃO RESPONDE	0									
RESPIRAÇÃ0	CAPAZ DE RESPIRAR PROFUNDAMENTE	2									
	DISPNEIA OU LIMITAÇÃO DA RESPIRAÇÃO	1									
	APNEIA	0									
SATURAÇÃO DE O2	CAPAZ DE MANTER SATURAÇÃO DE O2> 92%, RESPIRANDO EM AR AMBIENTE	2									
	NECESSITA DE O2 PARA MANTER A SATURAÇÃO >90%	1									
	SATURAÇÃO DE O2< DE 90%, COM SUPLEMENTAÇÃO DE O2	0									
CIRCULAÇÃO	PA COM VARIAÇÃO DE ATÉ 20% DO NÍVEL PRÉ-ANESTÉISCO	2									
	PA COM VARIAÇÃO DE 20% A 49% DO NÍVEL PRÉ-ANESTÉISCO	1									
	PA COM VARIAÇÃO ACIMA DE 50% DO NÍVEL PRÉ-ANESTÉISCO	0									
ATIVIDADE MUSCULAR	MOVIMENTA OS QUATRO MEMBROS	2									
	MOVIMENTA 2 MEMBROS	1									
	INCAPAZ DE MOVER OS MEMBROS VOLUNTARIAMENTE OU SOB COMANDO	0									
TOTAL DE PONTOS	**OBS: DE 8 A 10 PONTOS, PACIENTE APTO PARA ALTA**	-									

8. DIAGNÓSTICOS DE ENFERMAGEM NO PÓS-OPERATÓRIO IMEDIATO NA RPA

DIAGNÓSTICOS DE ENFERMAGEM

[ ] CONFUSÃO AGUDA
[ ] DOR AGUDA
[ ] RISCO DE ASPIRAÇAO
[ ] NAÚSEA
[ ] HIPOTERMIA PERIOPERATÓRIA
[ ] INTEGRIDADE DA PELE PREJUDICADDA
[ ] RISCO DE QUEDA
[ ] RISCO DE INFECÇÃO DE SITIO CIRÚRGICO
[ ] RISCO DE SANGRAMENTO
[ ] PADRÃO RESPIRATÓRIO INEFICAZ
[ ] RISCO DE DESEQUILIBRIO DO VOLUME DE LIQUÍDOS
[ ] MOILIDADE NO LEITO PREJUDICADA
[ ] RISCO DE GLICEMIA INSTÁVEL
[ ] ELIMINAÇAO URINÁRIA PREJUDICADA

ASS OF ENFERMEIRA:

INTERVENÇÕES DE ENFERMAGEM

[ ] PORPORCIONAR TRANQUILIDADE E CONFORTO
[ ] ADMINISTRAR OXIGÊNIO UMIDIFICADO
[ ] AVALIAR OS NÍVEIS E SINAIS DA DOR
[ ] AQUECER COM COBERTORES, E /OU MANTA TÉRMICA
[ ] AVALIAR CONDIÇÕES DA FERIDA OPERATÓRIA E DA PELE DO PACIENTE
[ ] AVALIAR ELIMINACÕES POR DRENOS E CATETERES
[ ] REALIZAR MUDANÇA DE DECÚBITO
[ ] MANTER MACA TRAVADA COM GRADE ELEVADA E VIGILÂNCIA
[ ] MANTER CABEÇEIRA ELEVADA 30 º/45 º
[ ] OBSERVAR NÁUSEAS E VÔMITOS
[ ] ADMINISTRAR MÉDICAÇÃO CONFFORME PRESCRIÇÃO
[ ] OBSERVAR SINAIS VITAIS (PA, FC, SPO2)
[ ] OBSERVAR E COMUNICAR SINAIS DE RENTENÇÃO URINÁRIA
[ ] OBSERVAR E COMUNICAR SINAIS DE SANGRAMENTO
[ ] AVALIAR HGT AOS SINASI DE HIPO OU HIPER GLICEMIA E EM PACIENTES DIABÉTICOS.

CONTROLE DE LÍQUIDOS NA RPA

HORA	LÍQUIDOS ADMINISTRADOS	ML	HORA	LÍQUIDOS ELIMINADOS	ML
:			:		
:			:		
:			:		

:			:		

ADMINISTRAÇÃO DE HEMODERIVADOS NA RPA

HORA	TIPO	ID BOLSA	VOLUME	FC (BPM)	PA (MMHG)	SAT O2	TEMP º C	ASSINATURA
:								
:								

ANOTAÇÕES/INTERCORRÊNCIAS DE ENFERMAGEM

ASSINATURA

ALTA MÉDICA DA RPA

OBSERVAÇÕES

ASSINATURA/CARIMBO ANESTESISTA **HORA DA ALTA DA RPA:______________**

TRANSPORTE

PRONTUÁRIO COMPLETO ENTREGUE COM: FICHA DA ANESTESIA [ ] PRESCRIÇÃO MÉDICA [ ] SAEP[ ] EXAMES[ ]

SAÍDA DO CC ÀS: ____________________ **ENCAMINHADO POR:__________________________**
